# Plasmid-mediated regulation of chromosomal gene expression and virulence in *Chlamydia psittaci*

**DOI:** 10.1128/msphere.00507-26

**Published:** 2026-09-28

**Authors:** Yixin Mo, Buwei Wang, Junjun Jia, Xiaomin Lin, Lin Li, Xiaoxin Pan, Kensuke Shima, Chuan Wang, Chunfu Yang

**Affiliations:** 1 School of Public Health and Emergency Management, Southern University of Science and Technology 255310, Shenzhen, China; 2 School of Medicine, SUSTech Homeostatic Medicine Institute, Southern University of Science and Technology 255310, Shenzhen, China; 3 School of Basic Medicine, Hunan Provincial Key Laboratory for Special Pathogens Prevention and Control, Institute of Pathogenic Biology, Hengyang Medical College, University of South China 34706, Hengyang, Hunan, China; 4 Institute of Medical Microbiology, University of Lübeck 9191, Lübeck, Germany; Medical College of Wisconsin, Milwaukee, Wisconsin, USA

**Keywords:** *Chlamydia psittaci*, plasmid, Pgp3, Pgp4, pathogenesis

## Abstract

*Chlamydia psittaci* (*Cps*) causes psittacosis, a zoonotic disease characterized by severe pneumonia and systemic infection, yet its pathogenic mechanisms remain poorly understood. While plasmids are established virulence determinants in *C. trachomatis* (*Ct*) and *Chlamydia muridarum* (*Cm*), their role in *Cps* pathogenesis is unknown. Here, we generated a panel of *Cps* transformants with individual deletions of plasmid genes (pgp1–pgp8) and characterized *in vitro* and *in vivo*. Genetic analysis revealed that Pgp1 and Pgp2 are required for plasmid maintenance, whereas Pgp3–Pgp8 are dispensable. Transcriptomic and qRT-PCR analyses identified a subset of chromosomal genes regulated by the *Cps* plasmid, distinct from those described in *Ct* and *Cm*. Pgp4 functions as a central regulator of chromosomal gene expression; however, unlike *Ct* or *Cm*, Pgp4 does not affect pgp3 transcript levels but is required for Pgp3 protein expression, indicating post-transcriptional regulation. We further show that Pgp7 positively regulates plasmid-dependent gene expression, whereas Pgp6 and Pgp8 negatively regulate this process. Functionally, the plasmid is a determinant of virulence in a murine lung infection model. Pgp3 is essential for host inflammatory responses and bacterial virulence *in vivo*. Collectively, these findings demonstrate plasmid-mediated regulation in *Cps* is distinct from that in other chlamydial species and highlight species-specific regulatory mechanisms underlying chlamydial pathogenesis.

**IMPORTANCE:**

*Chlamydia psittaci* is a zoonotic pathogen that causes severe and fatal pneumonia in humans, yet its molecular pathogenesis remains poorly understood. Here, we generated a series of *Cps* transformants carrying individual ORF deletion and characterized their functions both *in vitro* and *in vivo*. We show that Pgp4 and Pgp7 positively regulate a subset of chromosomal genes, whereas Pgp6 and Pgp8 act as negative regulators. Unexpectedly, Pgp4 regulates the major virulence factor Pgp3 at a post-transcriptional level, revealing a previously unrecognized mode of plasmid control. These findings provide new insights into the roles of plasmid-encoded genes in *Cps* biology and pathogenesis.

## INTRODUCTION

*Chlamydia psittaci* (*Cps*) is the etiological agent of avian chlamydiosis and human psittacosis (1). It is a zoonosis that commonly infects birds with asymptomatic infections (2). However, the importance of *Cps* as a human pathogen is largely underestimated. Exposure to infected birds is considered the major risk factor for human infection (3) although human- to-human transmission has been recently reported (4). In addition, an increasing number of psittacosis cases have been reported in several European countries and China (5, 6), largely due to the application of metagenomic next-generation sequencing in clinical diagnosis (7). Notably, *Cps* has been increasingly identified as a cause of severe or even fatal community-acquired pneumonia, posing a growing threat to public health.

Most *Chlamydia* species possess a cryptic 7.5-kb plasmid (8), and its role in chlamydial pathogenesis was underappreciated until the transformation method was developed (9). The plasmid encodes eight open reading frames (ORFs) encoding proteins designated plasmid gene proteins 1 to 8 (Pgp1 to Pgp8). Pgp1, Pgp2, Pgp6, and Pgp8 are important for plasmid maintenance (10, 11). Pgp4 is a master positive regulator of plasmid-encoded Pgp3 and the chromosomal genes (10), whereas Pgp5 is a negative regulator of Pgp4-regulated genes (12). Pgp3 is a virulence factor in the pathogenesis of *Ct* and *Cm* (13–15). However, whether the plasmid of *Cps* plays a similar role in regulating virulence and pathogenesis remains largely unknown.

In this study, we generated a series of *Cps* transformants carrying mutations in individual plasmid ORFs and systematically characterized their functions both *in vitro* and *in vivo*. Our results reveal distinct roles of plasmid genes in plasmid maintenance, transcriptional regulation, and virulence during infection. Notably, the regulatory and pathogenic roles of plasmid genes in *Cps* differ from those previously described in *Ct* and *Cm*. These findings highlight the evolutionary divergence of plasmid-mediated regulatory networks among chlamydial species and underscore the importance of plasmid–chromosome coevolution in shaping the pathogenesis of host-dependent intracellular pathogens.

## RESULTS

### *Cps* plasmid is a determinant in a murine lung infection model

**Fig 1:**
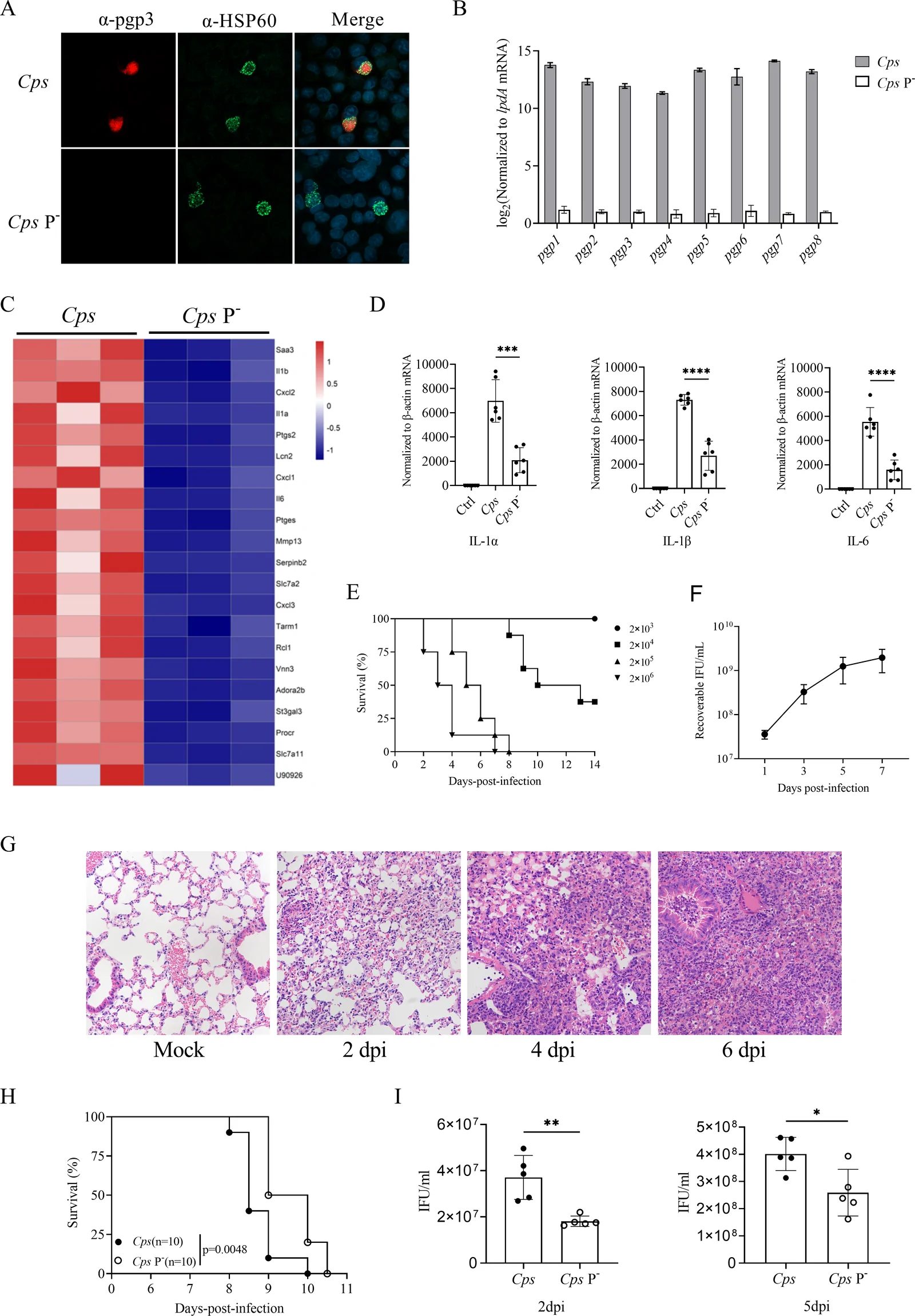
*Cps* plasmid-deficient strain is highly attenuated in a murine lung infection model. (**A**) A *Cps* plasmid-deficient strain was obtained through novobiocin treatment (125 μg/mL) followed by plaque purification. A549 cells infected with *Cps* and *Cps* P^−^ were stained for Pgp3 (red), Hsp60 (green), and DNA (blue) at 36 hpi. (**B**) qRT-PCR analysis of *Cps* and *Cps* P^−^ infected A549 cells confirmed complete loss of eight plasmid genes (pgp1–pgp8) in the *Cps* P^−^ strain, and *Cps* infection was included as a positive control. (**C**) Bulk RNA-seq of *Cps* and *Cps* P^−^-infected BMDMs at 24 hpi. BMDMs were infected with *Cps* and *Cps* P^−^ strains at a MOI of 1 (*n* = 3 per group). Total RNA was collected 24 hpi for RNA-seq analysis. The heatmap displays differentially expressed genes (|log₂FC| ≥ 1, adjusted *P* < 0.05), with red indicating upregulation and purple indicating downregulation. (**D**) qRT-PCR validation of IL-1α (**D**), IL-1β (**E**), IL-6 (**F**) expression in *Cps* and *Cps* P^−^-infected BMDMs. β-Actin was used as an internal control, and gene expression levels were normalized to the uninfected cells (two-tailed unpaired *t*-test). (**E**) LD₅₀ of *Cps* was determined in C57BL/6 mice following intratracheal infection. C57BL/6 mice (*n* = 8) were intratracheally inoculated with different dilutions of *Cps*. Mice were monitored for 14 days, and mortality rates were recorded. The LD₅₀ of *Cps* was calculated using the modified Karber method. (**F**) Bacterial burden in the lungs of infected mice at the indicated times post *Cps* infection. C57BL/6 mice were intratracheally inoculated with 1.5 LD₅₀ of *Cps*, and lung tissues were collected at 1, 3, 5, and 7 dpi (*n* = 3 per time point). Bacterial loads in lung homogenates were quantified by IFU/mL. (**G**) Histopathology of *Cps*-infected mice. C57BL/6 mice were intratracheally inoculated with 1.5 LD₅₀ of *Cps*. Histopathology of the lung at days 2, 4, and 6 dpi was characterized by severe leukocyte infiltration comprised primarily of neutrophils and macrophages. (**H**) Survival of mice infected with *Cps* and *Cps* P^−^ strains. C57BL/6 mice (*n* = 10/group) were intratracheally inoculated with 1.5 LD₅₀ of *Cps* or *Cps* P^−^ strains and observed for 11 days, and survival curves were plotted. The log-rank test revealed a significant difference between the two groups (*P* < 0.01). (**I**) Lung bacterial burden in mice infected with *Cps* and *Cps* P^−^ strains at 2 and 5 dpi. C57BL/6 mice (*n* = 5 per group) were intratracheally inoculated with 1.5 LD₅₀ of *Cps* and *Cps* P^−^. The lung was harvested at 2 and 5 dpi, and IFUs per lung were determined (two-tailed unpaired *t*-test). (**D, I**) Data are shown as mean ± SD. ****P < 0.0001, ***P < 0.001, **P < 0.01, *P < 0.05.

To investigate the contribution of the plasmid to *Chlamydia psittaci 6BC* (*Cps*) pathogenesis, we first generated a plasmid-deficient strain using novobiocin treatment (125 μg/mL) followed by plaque purification (16). Immunofluorescence staining confirmed the expression of the plasmid-encoded protein Pgp3 in the *Cps* strain, whereas Pgp3 was undetectable in the plasmid-deficient strain. In contrast, both strains expressed comparable Hsp60 (Fig. 1A). Consistently, qPCR analysis further confirmed that none of the eight ORFs were detectable in the cured strain (Fig. 1B), hereafter referred to as *Cps* P^−^.

We next examined whether the *Cps* plasmid affects host inflammatory responses. Mouse bone marrow-derived macrophages (BMDMs) were infected with either *Cps* or *Cps* P^−^ and subjected to transcriptomic profiling. RNA-seq analysis revealed a marked reduction in pro-inflammatory gene expression following infection with *Cps* P⁻, including IL-1α, IL-1β, and IL-6 (Fig. 1C). These results were validated by qRT-PCR, which confirmed significantly reduced cytokine expression in *Cps* P^−^-infected cells (Fig. 1D).

To assess plasmid-dependent virulence *in vivo*, we established a murine model of pulmonary infection. The median lethal dose (LD₅₀) of *Cps* was first determined in C57BL/6 mice via intratracheal inoculation. Mice were infected with increasing doses (2 × 10^3^ to 2 × 10⁶ IFU) and monitored for 14 days. All mice receiving 2 × 10⁵ or 2 × 10⁶ IFU succumbed to infection between 4- and 8-day post-infection (dpi). Mice receiving 2 × 10^4^ IFU showed 62.5% mortality (5/8), whereas no deaths occurred in the 2 × 10³ IFU group. The LD₅₀ was calculated to be 1.5 × 10⁴ IFU (Fig. 1E). Bacterial burden kinetics of *Cps* in the lung were assessed over time. *Cps* bacterial burden progressively increased over the course of infection, indicating active replication in the lung (Fig. 1F). Histopathologically, *Cps* infection caused extensive leukocyte infiltration comprised primarily of neutrophils and macrophages (Fig. 1G).

Next, C57BL/6 mice were intratracheally infected with 1.5 LD₅₀ of either *Cps* or *Cps* P^−^. Infection with the *Cps* led to rapid mortality, with all mice succumbing by 9–11 dpi. In contrast, mice infected with the *Cps* P^−^ strain exhibited significantly delayed mortality (Fig. 1H). Consistent with these observations, bacterial burdens in the lung at 2 and 5 dpi were significantly reduced in *Cps* P^−^-infected mice compared to those infected with the *Cps* strain (Fig. 1I). Together, these results indicate that the *Cps* plasmid plays a role in determining bacterial virulence.

### *Cps* plasmid transcriptionally regulates a different subset of chromosomal genes

**Fig 2:**
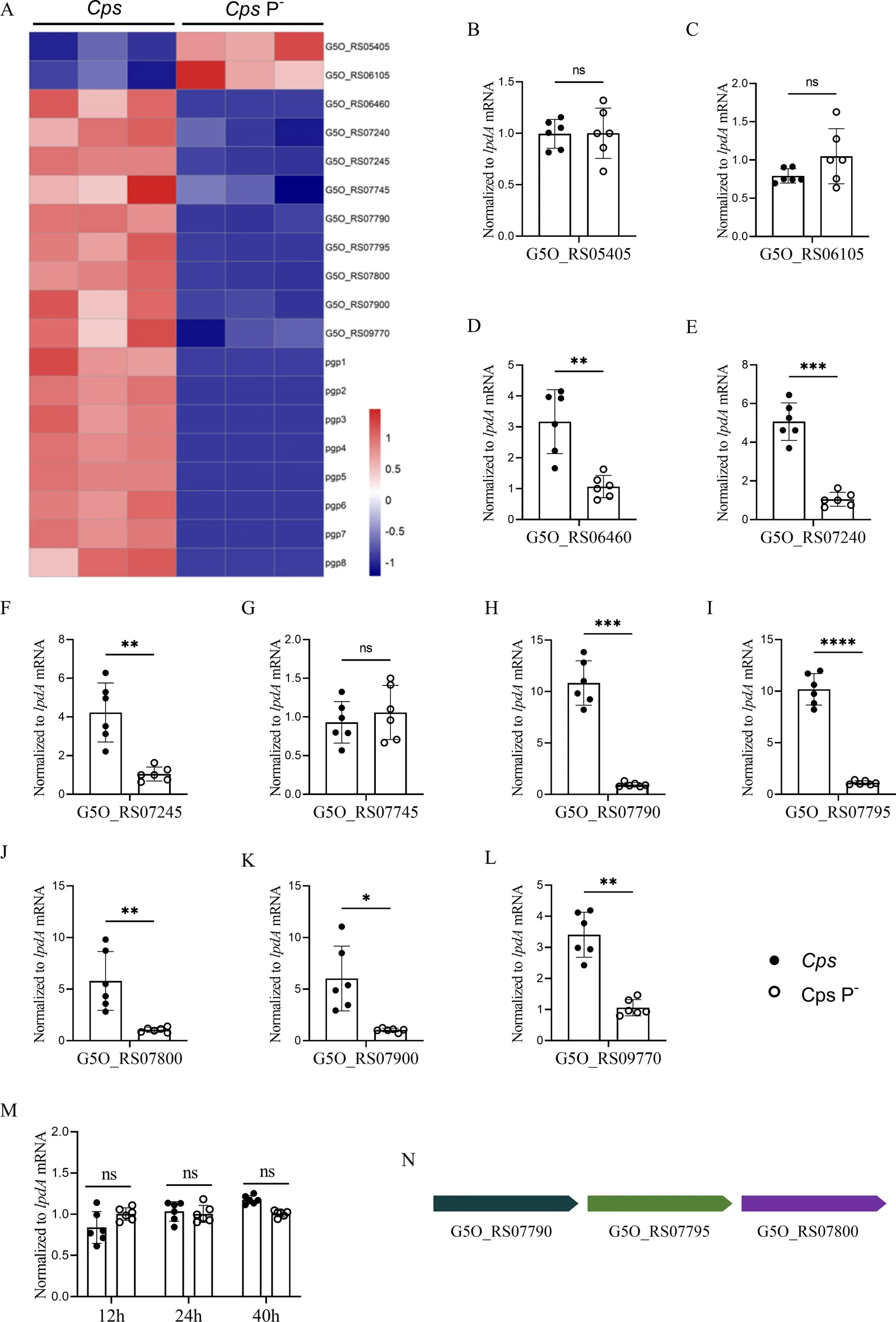
Transcriptional regulation of chromosomal genes by plasmid in *Cps*. (**A**) A549 cells were infected with *Cps* and *Cps* P^−^ at a MOI of 1 (*n* = 3 per group). Dual RNA-seq analysis was performed at 24 hpi. The heatmap displays differentially expressed genes in *Chlamydia* (|log_2_FC| ≥ 1, adjusted *P* < 0.05), with red indicating upregulation and blue indicating downregulation. *Z*-scores were calculated using *Z* = (*x −* μ)/σ, where *x* is the gene expression value, and μ and σ are the mean and standard deviation across samples for each gene. The results show that the expressions of 11 chromosomal genes were downregulated in *Cps* P^−^, eight plasmid genes (pgp1–pgp8) were absent in *Cps* P^−^. Note: *pgp1–pgp8* genes were transcriptionally undetectable based on raw expression levels in *Cps* P^−^. (**B–M**) Selected differentially expressed chromosomal genes in dual RNA-seq were validated by qRT-PCR. A549 cells were infected (MOI = 1, *n* = 3), and total RNA was collected at 24 hpi. Note: GlgA was not regulated by plasmid in *Cps*. Data are shown as mean ± SD. ****P < 0.0001, ***P < 0.001, **P < 0.01, *P < 0.05, ns not significant (two-tailed unpaired *t*-test). (**N**) Schematic diagram showing the arrangement of G50_RS07790, G50_RS07795, and G50_RS07800 on the *C. psittaci* genome. The direction of the arrows indicates the transcription direction; the three genes are arranged in a co-directional tandem configuration.

To determine whether the *Cps* plasmid was involved in regulating the expression of chromosomal genes, A549 cells were infected with *Cps* and *Cps* P⁻ strains, respectively, followed by dual RNA-seq analysis. Comparative transcriptomic profiling identified 19 differentially expressed transcripts, including 8 plasmid-encoded genes (Fig. 2A). To validate the RNA-seq results, qRT-PCR was performed on the differentially expressed genes. Consistent with the sequencing data, the expression levels of G5O_RS06460, G5O_RS07240, G5O_RS07245, G5O_RS07790, G5O_RS07795, G5O_RS07800, G5O_RS07900, and G5O_RS09770 were significantly reduced in *Cps* P⁻ compared to *Cps*, whereas the expression levels of genes G5O_RS05405, G5O_RS06105, and G5O_RS 07745 showed no significant differences between the two strains (Fig. 2B through L). Notably, GlgA, which is regulated by Pgp4 in *Ct* and *Cm*, did not exhibit any difference in *Cps* (Fig. 2M). Meanwhile, G5O_RS07790, G5O_RS07795, and G5O_RS07800 are arranged in a contiguous cluster on the genome (Fig. 2N).

Collectively, these results indicate that the *Cps* plasmid regulates the transcription of a specific subset of chromosomal genes, suggesting that plasmid regulation of chromosomal genes is species-specific.

### *Cps* ΔPgp6 and *Cps* ΔPgp8 are stably passaged in *Cps in vitro*

**Fig 3:**
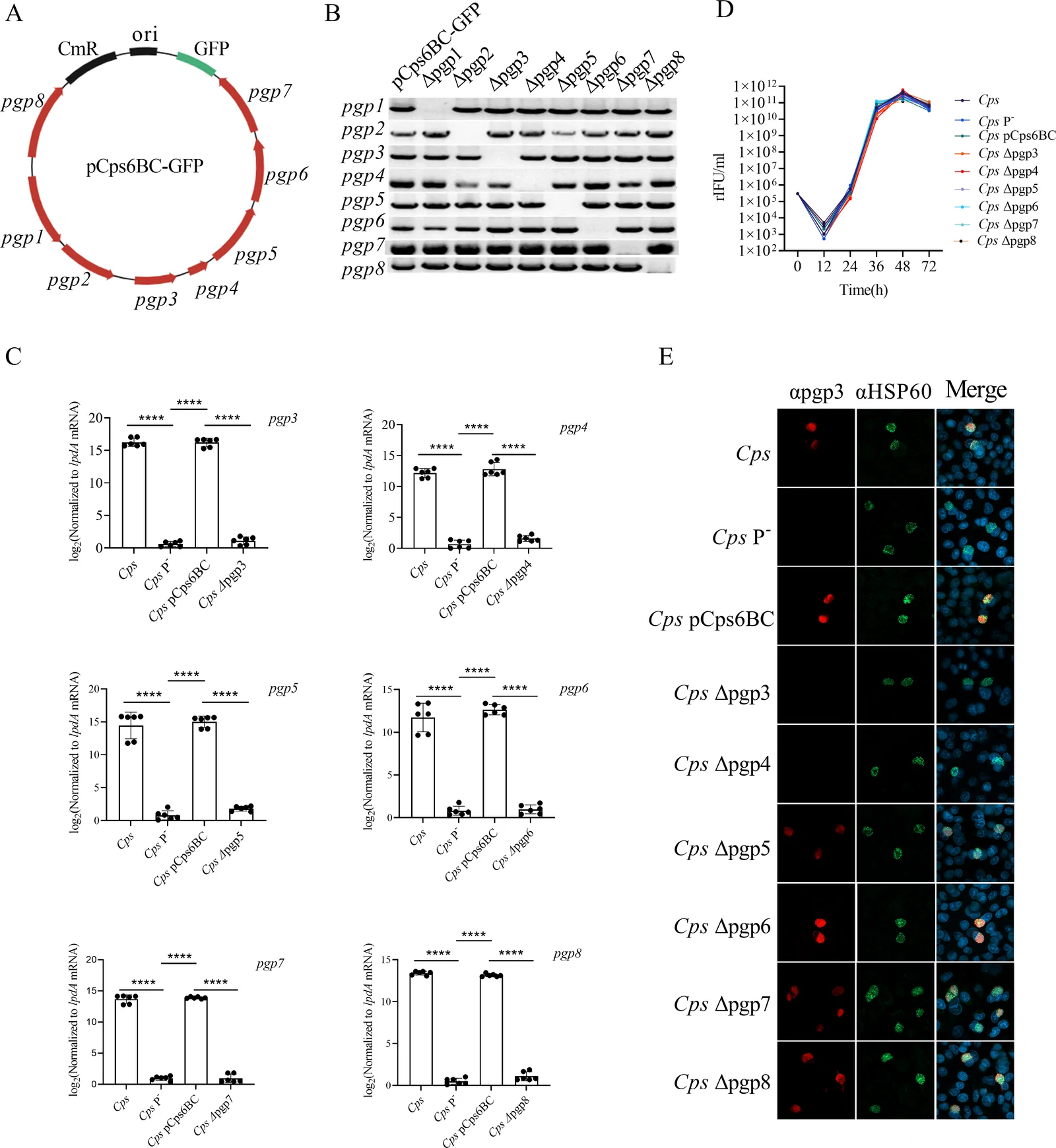
*Cps*Δpgp6 and *Cps*Δpgp8 were stably passaged in *Cps*. (**A**) Schematic of the pCps6BC-GFP shuttle vector. Eight open reading frames of *Cps* plasmid are shown in red, GFP is indicated, CmR and pUC origin (ori) are shown in black. The p*Cps*6BC sequence was amplified from *C. psittaci*, and an additional fragment was amplified from pCps-Tet-mCherry. (**B**) PCR amplification of the eight ORFs of pCps6BC and different ORF deletion plasmids. Compared to the full-length pCps6BC plasmid, each individual ORF knockout construct lacks the corresponding amplicon of expected size on agarose gels. (**C**) A549 cells were infected with *Cps*, *Cps* P^−^, and stable complemented strains for 24 h. Total DNA was extracted, and *pgp3–pgp8* were analyzed by qPCR to confirm each ORF deletion in complemented strains. Data are shown as mean ± SD. ****P < 0.0001 (two-tailed Mann–Whitney *U* test). (**D**) One-step growth curve of different *Cps* and its transformants. The pCps6BC-GFP shuttle vector and its derivatives were transformed into the plasmid-deficient strain *Cps* P^−^. *Cps* pCps6BC, *Cps* Δpgp3, *Cps* Δpgp4, *Cps* Δpgp5, *Cps* Δpgp6, *Cps* Δpgp7, and *Cps* Δpgp8 strains were obtained and stably passaged. A549 cells were infected at MOI = 1 with indicated strains. Cells were lysed at 12, 24, 36, 48, and 72 hpi, and progeny titers were determined on McCoy cells. No significant differences were observed among strains at any time point. (**E**) IFA staining of *Cps*, *Cps* P^−^, and stable complemented strains. A549 cells were infected with different strains for 40 h and stained for Pgp3 (red), Hsp60 (green), and DNA (blue), followed by confocal microscopy.

To investigate the contribution of plasmid-encoded genes to *Cps* biology, we generated a panel of plasmid deletion mutants. A recombinant shuttle vector was first constructed by replacing the penG cassette in pCps-Tet-mCherry (17) with a chloramphenicol resistance gene and substituting the backbone with the *Cps* 6BC plasmid, yielding pCps6BC-GFP (Fig. 3A). Using PCR-based deletion mutagenesis, individual deletions were introduced into each of the eight plasmid-encoded ORFs. Positive clones were screened by PCR with ORF-specific primers and verified by sequencing (Fig. 3B).

Previous studies have demonstrated that Pgp1, Pgp2, Pgp6, and Pgp8 are essential for plasmid maintenance in *Ct* and *Cm*. To assess whether this requirement is conserved in *Cps*, each deletion plasmid (pCps6BCΔPgp1–ΔPgp8) was transformed into the *Cps* P⁻ strain. The results showed that stable transformants were obtained following transformation with *Cps* ΔPgp3, *Cps* ΔPgp4, *Cps* ΔPgp5, *Cps* ΔPgp6, *Cps* ΔPgp7, and *Cps* ΔPgp8. In contrast, no stable transformants were recovered for ΔPgp1 or ΔPgp2. The genotypes of all recovered strains were confirmed by qPCR analysis (Fig. 3C). One-step growth curves revealed similar growth kinetics among all strains in A549 cells (Fig. 3D). Furthermore, IFA staining shows all stable transformants were Hsp60 positive, while Pgp3 was absent in *Cps* P⁻ strain, *Cps* ΔPgp3, and *Cps* ΔPgp4 (Fig. 3D).

Together, these results demonstrated that Pgp1 and Pgp2 are essential for plasmid maintenance in *Cps*. Notably, both Pgp6 and Pgp8 are dispensable in *Cps*, as *Cps* ΔPgp6 and *Cps* ΔPgp8 were stably maintained; however, we could not exclude the possibility that plasmid copy number and stability of Δpgp6 and Δpgp8 in the absence of antibiotic selection may vary. Overall, this divergence reveals fundamental species-specific differences in plasmid biology.

### Pgp4 and Pgp7 positively regulate, whereas Pgp6 and Pgp8 negatively regulate chromosomal gene expression in *Cps*

**Fig 4:**
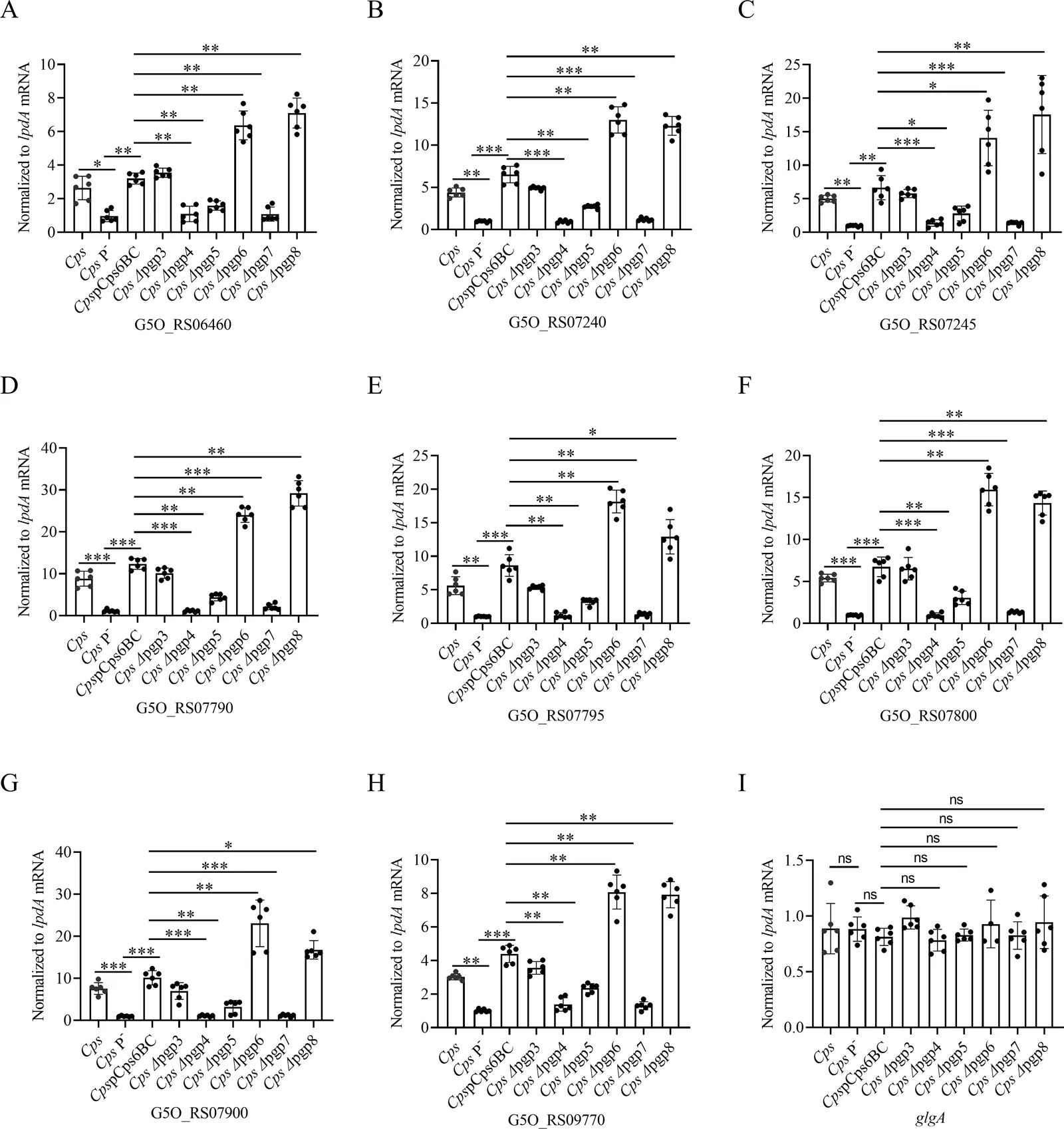
Pgp4 and Pgp7 positively regulate, whereas Pgp6 and Pgp8 negatively regulate chromosomal gene expression in *Cps*. (**A–H**) qRT-PCR analysis of plasmid-mediated regulation of chromosomal genes. A549 cells were infected with *Cps*, *Cps* P^−^, complemented strains, or individual ORF deletion mutants at MOI = 1. Total RNA was collected at 24 hpi. Transcription levels of G50_RS07790, G50_RS07795, G50_RS07800, G50_RS07900, G50_RS06460, G50_RS07240, and G50_RS07245 were measured using *lpdA* as the internal control. Data are shown as mean ± SD. ***P < 0.001, **P < 0.01, *P < 0.05 (two-tailed Mann–Whitney *U* test). (**I**) qRT-PCR analysis of *glgA* in A549 cells which were infected with *Cps*, *Cps* P^−^, complemented strains, or individual ORF deletion mutants at MOI = 1. Total RNA was collected at 24 hpi. Transcription levels of glgA were measured using *lpdA* as the internal control. (**A–I**) Data are expressed as mean ± SD. ***P < 0.001, **P < 0.01, *P < 0.05 (two-tailed unpaired *t*-test).

To further identify which plasmid-encoded factor(s) are responsible for regulating chromosomal gene regulation, we assessed gene expression by qPCR in *Cps*, *Cps* P⁻, *Cps* pCps6BC, and individual gene deletion mutants (*Cps* Δpgp3–Δpgp8). The identified plasmid-regulated chromosomal genes (G5O_RS06460, G5O_RS07240, G5O_RS07245, G5O_RS07790, G5O_RS07795, G5O_RS07800, G5O_RS07900, and G5O_RS09770) were analyzed. We found the expression profiles of the chromosomal genes in the *Cps* Δpgp4 mutant were highly similar to those in *Cps* P⁻, which is consistent with that in *Ct* and *Cm*. Strikingly, *Cps* Δpgp7 exhibited downregulation patterns similar to those observed in *Cps* P⁻ and *Cps* Δpgp4; *Cps* Δpgp5 showed twofold downregulation of these genes. In contrast, *Cps* Δpgp6 and *Cps* Δpgp8 showed significant upregulation of these genes. No significant changes were detected in *Cps* Δpgp3 mutant (Fig. 4A through H). However, whether these plasmid-encoded proteins directly modulate chromosomal transcription or instead exert indirect effects through alterations in plasmid physiology remains to be determined.

Glycogen synthase A (GlgA) is a conserved enzyme across chlamydial species that catalyzes glycogen synthesis within the inclusion lumen, thereby enabling intracellular carbon storage and supporting the developmental cycle. In *Chlamydia trachomatis*, GlgA expression is regulated by the plasmid-encoded protein Pgp4; however, the regulation of GlgA in *Cps* remains undefined. To address this gap, we investigated whether GlgA is also under plasmid-dependent regulation in *Cps*. Strikingly, glgA mRNA levels showed no significant difference between *Cps* and *Cps* P^−^, indicating that GlgA is not transcriptionally regulated by the plasmid in *Cps* (Fig. 4I).

Comparative genomic analyses have revealed both conservation and divergence between *Cps* and other chlamydial species (18–20). Although the overall genomic organization and plasmid gene content are conserved among chlamydiae, species-specific differences exist in host tropism, pathogenicity, and regulatory networks. Collectively, these findings suggest that plasmid-mediated regulation and plasmid–chromosome interactions may have evolved differently across chlamydial species, potentially contributing to their distinct pathogenic mechanisms.

### Pgp4 protein but not its RNA is required for Pgp3 expression

Pgp4 functions as a transcriptional activator of *pgp3*, and loss of Pgp4 results in a significant downregulation in *pgp3* transcript levels in *Ct* and *Cm*.

**Fig 5:**
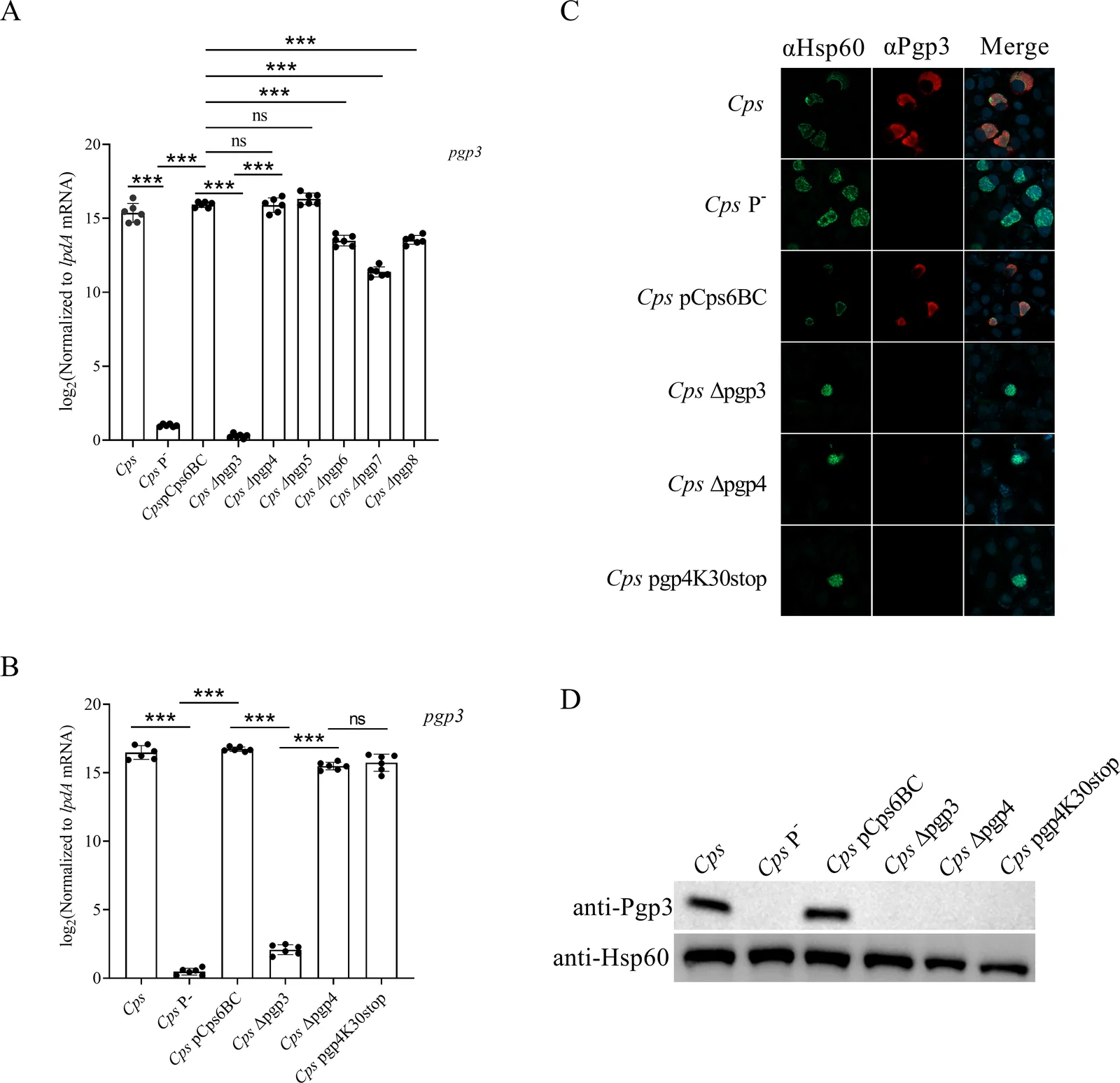
Pgp4 but not its RNA is required for Pgp3 expression. (**A**) qPCR analysis of relative mRNA expression levels of pgp3 in *Cps*, *Cps* P^−^, *Cps* pCps6BC, or individual ORF deletion mutants at MOI = 1. *lpdA* was included as the internal control. Data are shown as mean ± SD. ***P < 0.001, ns, not significant (two-tailed Mann–Whitney *U* test). (**B**) qPCR analysis of relative mRNA expression levels of pgp3 in *Cps*, *Cps* P^−^, *Cps* pCps6BC, *Cps*Δpgp4, and *Cps* pgp4K30stop at MOI = 1. *lpdA* was included as the internal control. Data are shown as mean ± SD. ***P < 0.001, ns, not significant (two-tailed Mann–Whitney *U* test). (**C**) IFA staining of *Cps*, *Cps* P^−^, *Cps* pCps6BC, *Cps* Δpgp3, *Cps* Δpgp4, and *Cps* pgp4K30stop, Pgp3 (red), Hsp60 (green), and DNA (blue), followed by confocal microscopy at magnification 63×. (**D**) Western blotting of *Cps*, *Cps* P^−^, *Cps* pCps6BC, *Cps* Δpgp4, and *Cps* pgp4K30stop.

In contrast, qPCR results showed that the mRNA level of *pgp3* in the *Cps* Δpgp4 mutant strain was comparable to those in *Cps* (Fig. 5A), whereas Pgp3 protein expression was substantially decreased (Fig. 3E).

To determine whether the loss of Pgp3 protein expression was attributable to the absence of Pgp4 function rather than a polarity effect caused by the Pgp4 gene deletion, we generated a mutant strain carrying a premature stop codon in *pgp4* (*Cps* pgp4K30stop). qPCR analysis showed that the mRNA level of Pgp3 in this mutant strain still did not differ significantly from that in the *Cps* (Fig. 5B). However, immunofluorescence and Western blot results demonstrated that Pgp3 protein expression was not restored (Fig. 5C and D). These results indicate that the absence of the Pgp3 protein is caused not by the deletion of the Pgp4 gene *per se* but rather by the absence of functional Pgp4 protein, indicating that Pgp4 regulates Pgp3 expression at the post-transcriptional level in *Cps*.

### Pgp3 is a determinant in a murine lung infection model

**Fig 6:**
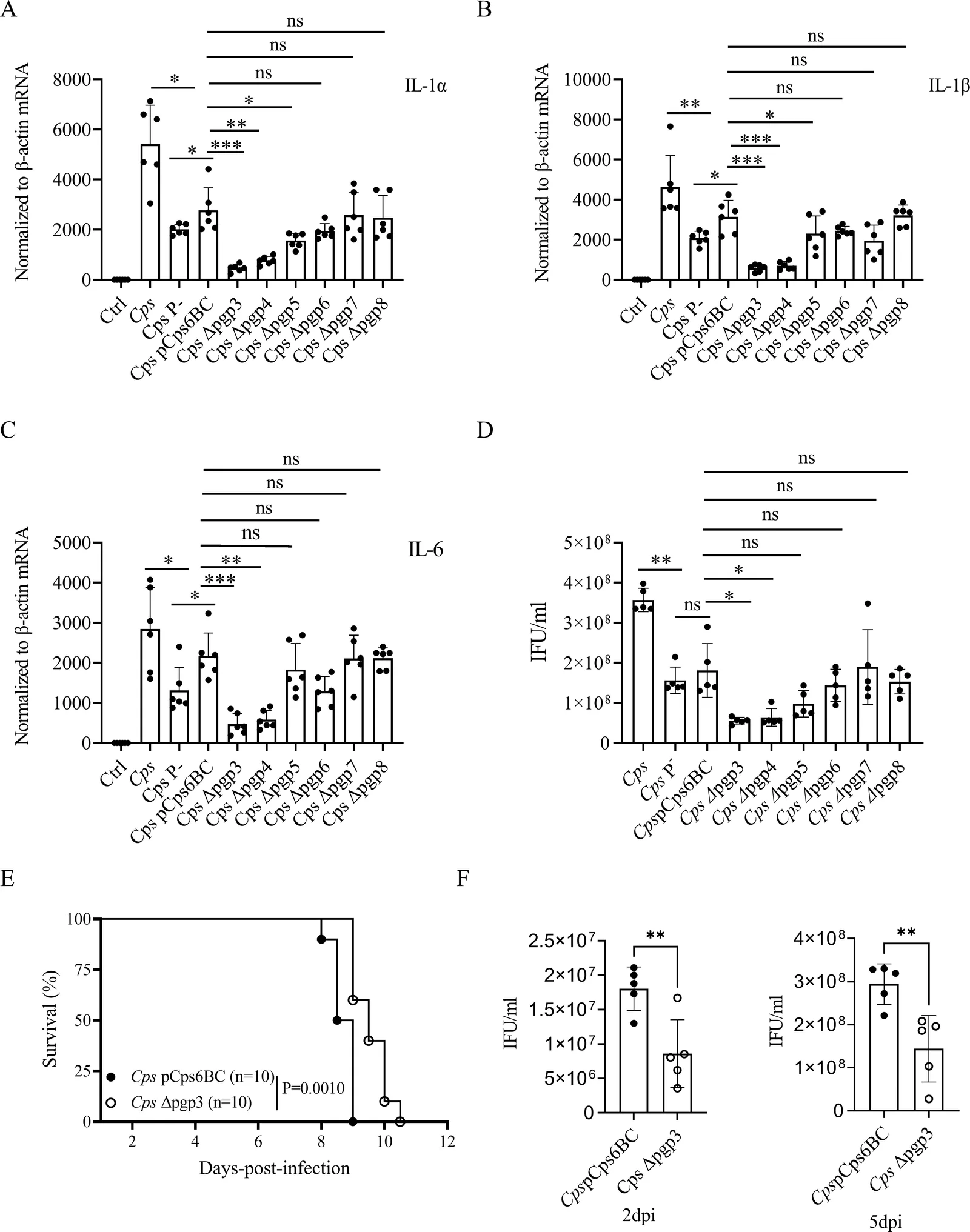
Inactivation of Pgp3 attenuated murine lung infection. (**A–C**) BMDMs were infected with *Cps*, *Cps* P^−^, complemented strains, or individual ORF deletion mutants at MOI = 1. Total RNA was collected at 24 hpi. IL-1α, IL-1β, and IL-6 mRNA levels were measured using β-actin as the internal control. Data are shown as mean ± SD. ***P < 0.001, **P < 0.01, *P < 0.05, ns, not significant (two-tailed Mann–Whitney *U* test). (**D**) C57BL/6 mice (*n* = 5 per group) were intratracheally infected with *Cps*, *Cps* P^−^, complemented strains, or individual ORF deletion mutants (2.5 × 10⁴ IFU/mouse). The lung was harvested at 2 dpi, and IFUs per lung were determined. Data are shown as mean ± SD. **P < 0.01, *P < 0.05, ns, not significant (two-tailed Mann–Whitney *U* test). (**E**) Survival of mice infected with *Cps* pCps6BC and *Cps*Δpgp3 strains. C57BL/6 mice (*n* = 10/group) were intratracheally inoculated with wild-type or plasmid-deficient strains (2.5 × 10⁴ IFU/mouse) and observed for 12 days, and survival curves were plotted. The log-rank test revealed a significant difference between the two groups (*P* < 0.001). (**F**) Lung bacterial burden in mice infected with *Cps* pCps6BC and *Cps*Δpgp3 strains at 2 and 5 days post-infection. C57BL/6 mice (*n* = 5 per group) were intratracheally inoculated with *Cps* pCps6BC and *Cps*Δpgp3 (2.5 × 10⁴ IFU/mouse). The lungs were harvested at 2 and 5 dpi, and IFUs per lung were determined. Data are shown as mean ± SD. **P < 0.01 (two-tailed unpaired *t*-test).

To evaluate the effects of plasmid-encoded proteins on host inflammatory responses and *in vivo* pathogenicity, we first measured the transcriptional levels of IL-1α, IL-1β, and IL-6 in BMDMs following infection with different strains. qRT-PCR analysis revealed that, compared with the plasmid-deficient strain and the wild-type, infection with *Cps* Δpgp3, *Cps* Δpgp4, and their complemented strains resulted in significantly reduced mRNA levels of all three cytokines (*P* < 0.01). In contrast, other deletion mutants (Δpgp5, Δpgp6, Δpgp7, Δpgp8) and their complemented strains showed no significant differences relative to the wild type (Fig. 6A through C).

Next, C57BL/6 mice were intratracheally infected with 1.5LD_50_, and bacterial burdens in the lungs were determined at day 2 dpi. The results showed that mice infected with *Cps* P^−^, *Cps* Δpgp3, or *Cps* Δpgp4 exhibited significantly lower pulmonary IFU compared with the *Cps*-infected group, whereas no significant differences were observed for the other strains (Fig. 6D).

Given the pronounced phenotype of the pgp3 deletion mutant both *in vitro* and *in vivo*, we further assessed its impact on host survival and bacterial burden dynamics. Mice infected with *Cps* Δpgp3 showed significantly prolonged survival compared to those infected with the complemented strain. Consistently, bacterial loads in the lungs at days 2 and 5 post-infection were markedly reduced in the *Cps* Δpgp3 group (Fig. 6E and F). Collectively, these findings showed Pgp3 is a virulent factor during *Cps* infection.

## DISCUSSION

In this study, we investigated the role of *Cps* plasmid in its pathogenesis. Byrne and colleagues reported that *Cps* plasmid does not significantly contribute to virulence in their infection model (21). In contrast, our data demonstrated the *Cps* plasmid, particularly Pgp3, is a virulent factor in our model. This discrepancy may be explained by differences in infection route, as intratracheal inoculation in our study more closely mimics natural psittacosis transmission via inhalation of contaminated aerosols from infected birds.

Pgp1 and Pgp2 are essential factors required for plasmid maintenance. Pgp1 exhibits sequence and functional homology to the DnaB family helicases of *Escherichia coli* and *Salmonella typhimurium*. DnaB functions as the essential replicative helicase that unwinds double-stranded DNA at the replication fork, thereby enabling coordinated leading- and lagging-strand synthesis. This homology suggests that Pgp1 may preserve conserved helicase-like structural features, including ATP-binding and hydrolysis motifs as well as domains involved in DNA strand separation, indicating a potential role in plasmid DNA metabolism and/or replication-associated processes critical for stable plasmid inheritance.

Pgp2 is the most conserved plasmid-encoded protein present across Chlamydia spp. Structural and functional analyses have identified a hairpin motif spanning residues 108–118 as essential for its DNA-binding activity (22). This region is, therefore, likely to mediate direct interactions with plasmid DNA, potentially contributing to plasmid replication, segregation, or the regulation of plasmid-encoded virulence determinants.

In *Ct*, *Cm*, and *C. caviae*, Pgp4 transcriptionally regulates both pgp3 and multiple chromosomal loci such as CT049–CT050, CT084, CT142–CT144, and CT798 (10, 11, 23, 24). In contrast, *Cps* Pgp4 regulates a distinct set of chromosomal genes, including G5O_RS06460, G5O_RS07240, G5O_RS07245, G5O_RS07790, G5O_RS07795, G5O_RS07800, G5O_RS07900, and G5O_RS09770. Notably, Pgp3 transcript levels were comparable to WT infection in both Δpgp4 and pgp4K30 stop mutants, but Pgp3 protein was absent in these two strains, suggesting a post-transcriptional regulatory mechanism distinct from that observed in other *Chlamydia* species. It has been reported that Pgp4 enhances the repressor function of chlamydia late regulator EUO (25).

Thus, Pgp4 may be involved in processes affecting Pgp3 stability or translation and could function as a chaperone to facilitate Pgp3 trimerization and its integration into the outer membrane in *Cps*. The outer membrane-associated Pgp3 is required for *Cm* to overcome gastric acid, whereas secreted Pgp3 facilitates the bacterial traverse of the ileocecal barrier to reach the large intestine (26–28). *Cps* persists in the wild birds (29), and human infection occurs in the respiratory tract following inhalation of aerosolized avian feces. Therefore, enhanced Pgp4 function may promote gut persistence of *Cps*.

*Cps* Pgp4-regulated chromosomal genes lack homologs in other chlamydial species but share sequence similarity with genes from *C. abortus* and *C. suis*, suggesting species-specific adaptation. However, *Cps* Δpgp4 exhibits similar bacterial burden or mortality compared to *Cps* Δpgp3, indicating these Pgp4-regulated chromosomal genes do not function in mammal hosts. Given that *Cps* primarily infects avian hosts, we speculate that these Pgp4-regulated genes could function in evasion of the avian immune response and contribute less to virulence in mammalian systems.

Pgp6 is a partial homolog of *E. coli* SopA/SopB and ParA/ParB systems and functions in plasmid partitioning and copy number control, which is essential for plasmid maintenance in *Ct* and *Cm* (10, 11). In contrast, we successfully generated a *Cps* Δpgp6 transformant that was stably passaged, indicating that Pgp6 is not essential for plasmid maintenance in *Cps*. Interestingly, the Pgp6 ORF has a homolog in the chromosome (G5O_RS09950, accession number: NC_017287.1) which is present in all chlamydiae including those lacking of plasmid. We reason that the *Cps* chromosomal gene G5O_RS09950 is functionally redundant when Pgp6 is inactive, why the homolog of Pgp6 in the genome of *Ct* or *Cm* cannot function a redundant role needs to be studied.

Pgp7 gene, which shares homology to integrase (30), is dispensable for plasmid maintenance. Naturally occurring *Ct* variants harboring disruptions in pgp7 have been identified, including a Swedish variant carrying a 377-bp deletion that rapidly spread in the population (31). Consistent with this, inactivation of pgp7 in *Ct*, *Cm,* or *Cps* does not result in detectable growth defects *in vitro*. Compared with *Cps*, a 200-nt deletion within Pgp7 (CDS1) of the pCpnE1 plasmid has been reported (32); similarly, Pgp7 in *Ct* and *Cm* is truncated at the C terminus by 140 bp. Our results show that inactivation of pgp7 in *Cps* leads to significant downregulation of a subset of Pgp4-controlled chromosomal genes but not including Pgp3, which is nonfunctional in *Ct* or *Cm*. This suggests that Pgp7 may complement the function of Pgp4 in *Cps*, highlighting the species-specific role of the plasmid in *Cps* biology.

Pgp8, which exhibits homology to recombinase/integrase, is indispensable for plasmid maintenance in *C. trachomatis* and *C. muridarum*. In contrast, we found that inactivation of Pgp8 does not impair plasmid maintenance in *Cps*. More importantly, *Cps* Pgp8 negatively regulates a subset of Pgp4-controlled chromosomal genes at the transcriptional level, indicating a species-specific regulatory function distinct from that observed in other chlamydial species. Notably, naturally occurring *Cps* HJ strain isolates lacking both Pgp7 and Pgp8 have been identified in meat pigeons exhibiting severe respiratory distress and high mortality. This observation is consistent with our findings that Pgp8 represses Pgp4-controlled gene expression (33, 34).

Notably, structural prediction revealed high per-residue confidence (>90%) between *Cps* Pgp7 and Pgp8 and members of the tyrosine recombinase family, including proteins from *Bacillus subtilis*, *Shigella flexneri*, and *Salmonella bongori* (AlphaFold3). Tyrosine recombinases are known to mediate site-specific DNA recombination, including promoter rearrangements and reversible gene activation states. Together, these features raise the possibility that Pgp7 may function as a DNA-modifying regulator that influences chromosomal gene accessibility or promoter architecture. Therefore, we propose that Pgp7 acts downstream of Pgp4 as a modulatory regulator that refines chromosomal gene expression, potentially through recombination-associated modulation of transcriptional states. In contrast, Pgp6 and Pgp8 may act as negative regulators, providing a counterbalancing mechanism to restrain chromosomal gene expression.

This model further raises the question of why Pgp6, Pgp7, and Pgp8 in *Ct* and *Cm* do not appear to exhibit similar functions. One possible explanation is that, during host adaptation and shifts in tissue tropism, *Chlamydia* spp. have undergone divergent selective pressures that reshaped plasmid-mediated regulatory networks. The observed divergence in sequence conservation and regulatory outputs of Pgp6–Pgp8 between *Cps* and other chlamydial spp. support this hypothesis (34). However, this model remains speculative and requires further experimental validation.

Given the significant economic impact of animal chlamydial infections on livestock and wildlife (35), our findings provide a new framework for investigating other animal chlamydiae, such as *C. abortus*, *C. pecorum*, and *C. suis*. This approach could prove valuable for the development of species-specific attenuated vaccines and plasmid-based diagnostic markers. Future comparative studies across different *Chlamydia* species will be crucial for a better understanding of the evolutionary plasticity in plasmid-mediated virulence regulation.

## MATERIALS AND METHODS

### Chlamydial strains

The plasmid-free strain (*Cps* P^−^) was derived by novobiocin treatment as previously described. All strains were plaque cloned, propagated in McCoy cells, purified, and whole-genome sequenced. *Cps* 6BC strain was also whole-genome sequenced. No chromosomal SNP differences were detected between the two strains. However, compared with the reference genome NC_017287.1, both strains shared multiple identical single-nucleotide polymorphisms (SNPs) (36). These SNPs have been included in Table S3. Dulbecco’s modified Eagle medium (DMEM-10) supplemented with high glucose, 10% fetal bovine serum, and gentamicin (10 μg/mL) were used for cell culture.

### Generation of *Cps* transformants

The plasmid shuttle vector pCps6BC-GFP was derived from pCps-Tet-mCherry by deleting the tet promoter and mCherry. To obtain different ORF deletion mutants, DNA fragments lacking each ORF were amplified by PCR and ligated to shuttle vector pCps6BC-GFP using In-Fusion HD cloning. The primer pairs information was listed in Table S4.

Briefly, PCR conditions were as follows: initial denaturation at 95°C for 3 min, followed by 35 cycles per kb at 98°C for 10 s, 55°C for 5 s, 68°C for 10 s, and a final extension at 68°C for 5 min (Toyobo, KOD One TM PCR Master Mix-Blue). PCR products were then purified using the Novozymes PCR Purification Kit and digested with DpnI to remove the template DNA. The fusion products were transformed into GM2163 competent cells using the In-Fusion HD Cloning Kit (Biosharp), screened on chloramphenicol-resistant (50 μg/mL) LB agar plates, and verified by Sanger sequencing.

To generate different *Cps* transformants, *Cps* P^−^ strain was transformed with each expression construct to obtain the transformants *Cps* pCps6BC, *Cps* Δpgp3, *Cps* Δpgp4, *Cps* Δpgp5, *Cps* Δpgp6, *Cps* Δpgp7, *Cps* Δpgp8 as previously described (9). Briefly, 1 × 10^8^ IFU *Cps* and 6 µg of plasmid DNA were mixed in a total volume of 250 μL of CaCl_2_ buffer and incubated at room temperature for 30 min. The mixture was then added to 18 mL of DMEM pipetted into single wells of a six-well plate (3 mL per well) and centrifuged at 545*g* for 1 h at room temperature. Infected cells were first supplemented with fresh DMEM-10% FBS containing gentamicin (10 μg/mL) for 10 h and changed with fresh DMEM-10% FBS containing gentamicin (10 μg/mL) and chloramphenicol (0.5 μg/mL) for selection for additional 32 h of culture. The cells were harvested at 44 hours post-infection (hpi) with 1 mL filter tips and vortexed with glass beads. The cell debris was removed by spinning at 1,000 rpm for 5 min. The supernatant was termed passage 0 (P0). P0 can be stored at −80°C freezer or directly for P1 infection. Fluorescence positive transformants were usually got at P2 or P3. All the transformants were Sanger sequence verified, plaque cloned, propagated in McCoy cells, and purified as previously described (37).

### Murine model of intratracheal infection

Six- to eight-week-old female C57BL/6 mice were purchased from Guangdong GemPharmatech Co., Ltd. Mice were maintained in the Animal Center of the University of South China.

Six- to eight-week-old mice were anesthetized with isoflurane and fixed on an oblique plane. The tongue of each mouse was pulled out, and the throat was illuminated with a focused cold light lamp; a small venous catheter with a microliter syringe 22G was directly inserted into the trachea. Bacterial suspension was applied to the microliter syringe and was inhaled by the mice. Mice were monitored for survival up to 21 days post infection (dpi).

To determine the bacterial burden in the lung, infected mice were euthanized at 2 or 5 dpi, and lungs were harvested and homogenized. The supernatant of homogenized samples was further inoculated into McCoy cells for enumeration of recoverable IFU (rIFU).

### Dual RNA-seq

A549 cells (1.2 × 10^6^ cells per well) were infected with *Cps* and *Cps* P^−^ at a multiplicity of infection (MOI) of 1 with 3 replicates per infection. The infected cells were harvested at 24 hpi, and total RNA was isolated using TRIzol Reagent (Thermo Scientific, cat. #15596026). Genomic DNA was then removed using DNase I (NEB, cat. #M0303L). RNA quality and quantification were performed by using LabChip GX Touch system (Revvity) and RNA Broad Range Assay kit (Thermo Scientific, cat. #Q10210), respectively.

rRNA was first depleted using Ribo-off rRNA Depletion Kit (Vazyme, China, cat. #N406 for animal, cat. #N409 for plant, cat. #N417 for bacteria), and 2 µg total RNA was then used for stranded RNA sequencing library preparation using KC Digital Stranded mRNA Library Prep Kit (Seqhealth Co., Ltd. Wuhan, China) according to manufacturer’s instructions. The kit eliminates duplication bias and errors in PCR and sequencing steps, by using unique molecular identifier (UID) of 12 random bases to label the pre-amplified cDNA molecules. PCR products corresponding to fragments ranging from 200 to 500 bp were enriched, quantified, and sequenced on a Novaseq X Plus (Illumina) platform using the PE150 mode to generate paired-end reads.

Raw sequencing data were first filtered by fastp (version 0.23.0) and trimmed to remove low-quality reads and adaptor sequences. Clean reads were further clustered to eliminate any errors or biases according to the Unique Molecular Identifier (UMI) sequences. The deduplicated reads were mapped to the reference genome of A549 human and *Cps* 6BC using STAR software (version 2.5.3a) with default parameters.

The reads mapped to the exon regions of each gene were counted by featureCounts (Subread-1.5.1; Bioconductor), and then, RPKM was calculated. Genes differentially expressed between groups were identified using the edgeR package (version 3.28.1). Genes with an adjusted *P* value <0.05 and fold change >2 were considered significantly differentially expressed.

### Quantitative real-time RT-PCR

A549 or BMDM cells (1.2 × 10^6^ cells per well) were infected with different *Cps* strains at a MOI of 1 with 3 replicates per infection. The infected cells were harvested at 24 hpi, and total RNA was extracted using AG RNAex Pro Reagent (Accurate, AG21102). One microgram of RNA was reverse-transcribed into cDNA using Evo M-MLV RT Kit with gDNA Clean (AG11711). qPCR was performed with SYBR Green Pro Taq HS Premix (AG11701) according to the manufacturer’s instructions. The *lpdA* gene, which encodes dihydrolipoyl dehydrogenase, was used as an internal reference gene as described previously (10–12). Fold changes were determined using 2^(−ΔΔCt)^ method. Primer sequences are listed in Table S1, and their specificity had been confirmed using Primer-BLAST (NCBI).

### Generation of rabbit/mouse polyclonal antibodies

pET28a-Hsp60 and pET28a-Pgp3 were constructed for purification of recombinant *Cps* Hsp60 and *Cps* Pgp3 proteins. The plasmids were then transformed into *E. coli* BL21(DE3) and induced with 0.1 mM IPTG at 25°C for 16 h; the 6× His fusion proteins were further purified by Ni-NTA chromatography and ion exchange column chromatography.

Five hundred micrograms of recombinant protein *Cps* Hsp60 in 500 μL 1× PBS was mixed with 500 μL of MF59 adjuvant (MF220221, RVT) and subcutaneously injected into New Zealand white rabbit. Twenty micrograms of recombinant protein *Cps* Pgp3 in 100 μL 1× PBS was mixed with 100 μL of MF59 adjuvant and intramuscularly injected into BALB/c mice. Rabbit or mice were then boosted twice intramuscularly at weeks 4 and 8 with the aforementioned proteins or plasmid in MF59 adjuvant. Sera were collected at 2 weeks after the third immunization.

### Immunofluorescence

McCoy cells were cultured on glass coverslips in 24-well plates and infected with different *Cps* strains at MOI = 0.1 for 36 h. The infected cells were fixed with cold methanol for 10 min. Mouse anti-Pgp3 mouse polyclonal antibody (1:1,000) and rabbit anti-Hsp60 polyclonal antibody (1:2,000) were used for primary staining; goat anti-mouse IgG secondary antibody conjugated with DyLight 594 (Abbkine) and goat anti-rabbit IgG conjugated with FITC (Thermo Fisher) were at 1:1,000. Coverslips were further stained with 4′,6′-diamidino-2-phenylindole (DAPI) at a dosage of 1:1,000 for 5 min in PBS and mounted with ProLong gold. Images were acquired using a Zeiss LSM 900 confocal microscopy and analyzed using Zen microscopy software.

### Statistical analyses

GraphPad Prism 9.0 software was used for data analysis. Statistical parameters including the exact value of *n* (mean ± SD) and statistical significance are described in the figures. All the graphics used in the figures have been created by the authors. No images have been sourced from databases.

## Data Availability

The RNA-sequencing data generated in this study have been deposited in the NCBI Sequence Read Archive (SRA) under BioProject accession number PRJNA1523087.

## SUPPLEMENTAL MATERIAL

Supplemental captions msphere.00507-26-s0001.docx Captions for Tables S1 to S4.

Table S1 msphere.00507-26-s0002.xlsx RNA-seq analysis of *Cps*- and *Cps* P^−^-infected BMDM.

Table S2 msphere.00507-26-s0003.xlsx RNA-seq analysis of *Cps*- and *Cps* P^−^-infected A549 cells.

Table S3 msphere.00507-26-s0004.xlsx Whole genome sequence of *Cps* and *Cps* P^−^.

Table S4 msphere.00507-26-s0005.xlsx Primers used in this study.

ASM does not own the copyrights to Supplemental Material that may be linked to, or accessed through, an article. The authors have granted ASM a non-exclusive, world-wide license to publish the Supplemental Material files. Please contact the corresponding author directly for reuse.
