# Supplementary material for: Plasmid-mediated regulation of chromosomal gene expression and virulence in Chlamydia psittaci
Source: mSphere. 2026 Sep 28;11(10):e00507-26. doi: 10.1128/msphere.00507-26 (PMC13711795; doi:10.1128/msphere.00507-26)
Supplement: Supplementary file 1 [file msphere.00507-26-s0001.docx]

**Supplemental Table S1. NA-sequencing analysis of *Cps*- and *Cps* P^-^-infected BMDM.** Murine bone marrow-derived macrophages (BMDMs) were infected with *C. psittaci* (*Cps*) or the *Cps* P⁻ strain at a multiplicity of infection (MOI) of 1 (n = 3 per group). Total RNA was extracted at 24 h post-infection (hpi) and subjected to RNA-sequencing analysis. Ribosomal RNA was depleted, followed by cDNA library preparation and sequencing on an Illumina platform. Raw sequencing reads were quality-filtered and trimmed to remove low-quality sequences and adapter contamination. The clean reads were aligned to the mouse reference genome using STAR (version 2.5.3a) with default parameters. Gene expression levels were quantified as reads per kilobase of transcript per million mapped reads (RPKM). Differentially expressed genes (DEGs) between groups were identified using the edgeR package (version 3.28.1). Genes with an adjusted P value < 0.05 and a Log_2_ fold change > 2 was considered significantly differentially expressed.

**Supplemental Table S2. RNA seq analysis of *Cps*- and *Cps* P^-^-infected A549 cells.** A549 cells were infected with *C. psittaci* (*Cps*) or the *Cps* P⁻ strain at a multiplicity of infection (MOI) of 1 (n = 3 per group). Total RNA was extracted at 24 h post-infection (hpi) and subjected to RNA-sequencing analysis. Ribosomal RNA was depleted, followed by cDNA library preparation and sequencing on an Illumina platform. Raw sequencing reads were quality-filtered and trimmed to remove low-quality sequences and adapter contamination. The clean reads were aligned to the *Cps* 6BC reference genome (NC_017287.1) using STAR (version 2.5.3a) with default parameters. Gene expression levels were quantified as reads per kilobase of transcript per million mapped reads (RPKM). Differentially expressed genes (DEGs) between groups were identified using the edgeR package (version 3.28.1). Genes with an adjusted P value < 0.05 and a Log_2_ fold change > 2 was considered significantly differentially expressed.

**Supplemental Table S3. Whole genome sequence of *Cps* and *Cps* P^-^.**

Genomic DNA from *C. psittaci* (*Cps*) and the *Cps* P⁻ strains was isolated from purified elementary bodies (EBs). Whole-genome sequencing was performed by Novogene Co., Ltd. The genome sequences of *Cps* and *Cps* P⁻ were compared with each other and aligned against the reference genome (*C. psittaci* strain 6BC; NC_017287.1). No chromosomal single-nucleotide polymorphism (SNP) differences were identified between the two strains. Compared with the reference genome NC_017287.1, both strains harbored the same set of 24 chromosomal SNPs.

**Supplement material 4. Primers used in this study.** Primer sequences used for plasmid ORF cloning, qRT-PCR analysis, and generation of Hsp60- and Pgp3-expression constructs are listed.
